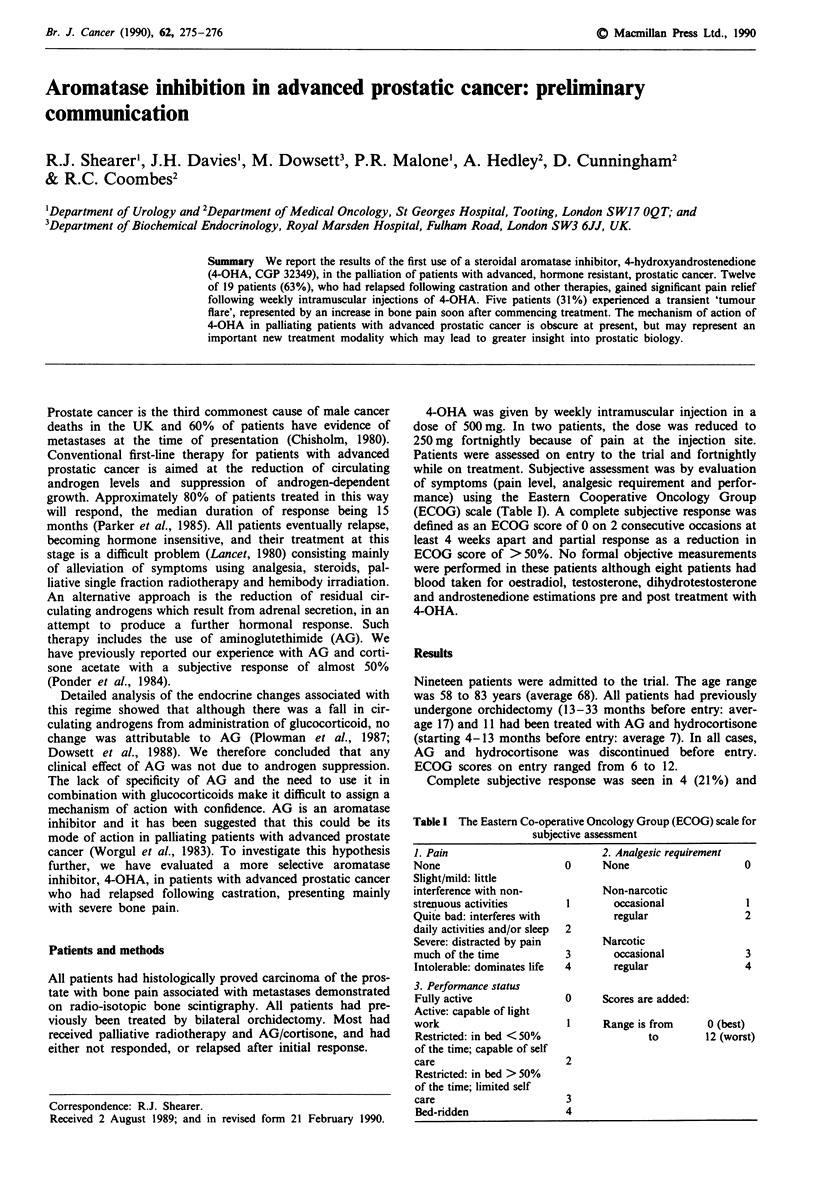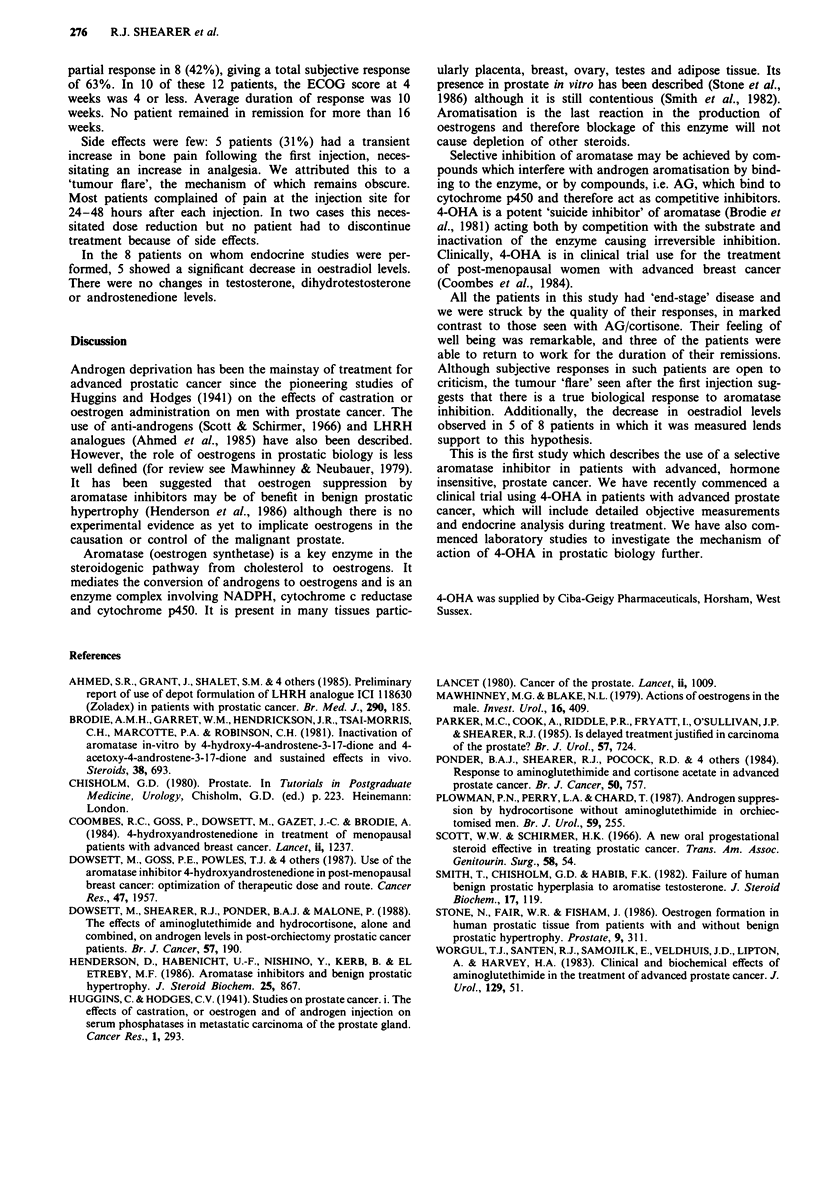# Aromatase inhibition in advanced prostatic cancer: preliminary communication.

**DOI:** 10.1038/bjc.1990.276

**Published:** 1990-08

**Authors:** R. J. Shearer, J. H. Davies, M. Dowsett, P. R. Malone, A. Hedley, D. Cunningham, R. C. Coombes

**Affiliations:** Department of Urology, St Georges Hospital, Tooting, London, UK.

## Abstract

We report the results of the first use of a steroidal aromatase inhibitor, 4-hydroxyandrostenedione (4-OHA, CGP 32349), in the palliation of patients with advanced, hormone resistant, prostatic cancer. Twelve of 19 patients (63%), who had relapsed following castration and other therapies, gained significant pain relief following weekly intramuscular injections of 4-OHA. Five patients (31%) experienced a transient 'tumour flare', represented by an increase in bone pain soon after commencing treatment. The mechanism of action of 4-OHA in palliating patients with advanced prostatic cancer is obscure at present, but may represent an important new treatment modality which may lead to greater insight into prostatic biology.


Br. J. Cancer (1990), 62, 275-276 © Macmillan Press Ltd., 1990

### Aromatase inhibition in advanced prostatic cancer: preliminary communication

R.J. Shearer[1], J.H. Davies[1], M. Dowsett[3], P.R. Malone[1], A. Hedley[2], D. Cunningham[2] & R.C. Coombes[2]

[1]*Department of Urology and* [2]*Department of Medical Oncology, St Georges Hospital, Tooting, London SW17 0QT; and* [3]*Department of Biochemical Endocrinology, Royal Marsden Hospital, Fulham Road, London SW3 6JJ, UK.*

**Summary** We report the results of the first use of a steroidal aromatase inhibitor, 4-hydroxyandrostenedione (4-OHA, CGP 32349), in the palliation of patients with advanced, hormone resistant, prostatic cancer. Twelve of 19 patients (63%), who had relapsed following castration and other therapies, gained significant pain relief following weekly intramuscular injections of 4-OHA. Five patients (31%) experienced a transient 'tumour flare', represented by an increase in bone pain soon after commencing treatment. The mechanism of action of 4-OHA in palliating patients with advanced prostatic cancer is obscure at present, but may represent an important new treatment modality which may lead to greater insight into prostatic biology.

Prostate cancer is the third commonest cause of male cancer deaths in the UK and 60% of patients have evidence of metastases at the time of presentation (Chisholm, 1980). Conventional first-line therapy for patients with advanced prostatic cancer is aimed at the reduction of circulating androgen levels and suppression of androgen-dependent growth. Approximately 80% of patients treated in this way will respond, the median duration of response being 15 months (Parker *et al.*, 1985). All patients eventually relapse, becoming hormone insensitive, and their treatment at this stage is a difficult problem (*Lancet*, 1980) consisting mainly of alleviation of symptoms using analgesia, steroids, palliative single fraction radiotherapy and hemibody irradiation. An alternative approach is the reduction of residual circulating androgens which result from adrenal secretion, in an attempt to produce a further hormonal response. Such therapy includes the use of aminoglutethimide (AG). We have previously reported our experience with AG and cortisone acetate with a subjective response of almost 50% (Ponder *et al.*, 1984).

Detailed analysis of the endocrine changes associated with this regime showed that although there was a fall in circulating androgens from administration of glucocorticoid, no change was attributable to AG (Plowman *et al.*, 1987; Dowsett *et al.*, 1988). We therefore concluded that any clinical effect of AG was not due to androgen suppression. The lack of specificity of AG and the need to use it in combination with glucocorticoids make it difficult to assign a mechanism of action with confidence. AG is an aromatase inhibitor and it has been suggested that this could be its mode of action in palliating patients with advanced prostate cancer (Worgul *et al.*, 1983). To investigate this hypothesis further, we have evaluated a more selective aromatase inhibitor, 4-OHA, in patients with advanced prostatic cancer who had relapsed following castration, presenting mainly with severe bone pain.

### Patients and methods

All patients had histologically proved carcinoma of the prostate with bone pain associated with metastases demonstrated on radio-isotopic bone scintigraphy. All patients had previously been treated by bilateral orchidectomy. Most had received palliative radiotherapy and AG/cortisone, and had either not responded, or relapsed after initial response.

Correspondence: R.J. Shearer.
Received 2 August 1989; and in revised form 21 February 1990.

4-OHA was given by weekly intramuscular injection in a dose of 500 mg. In two patients, the dose was reduced to 250 mg fortnightly because of pain at the injection site. Patients were assessed on entry to the trial and fortnightly while on treatment. Subjective assessment was by evaluation of symptoms (pain level, analgesic requirement and performance) using the Eastern Cooperative Oncology Group (ECOG) scale (Table I). A complete subjective response was defined as an ECOG score of 0 on 2 consecutive occasions at least 4 weeks apart and partial response as a reduction in ECOG score of >50%. No formal objective measurements were performed in these patients although eight patients had blood taken for oestradiol, testosterone, dihydrotestosterone and androstenedione estimations pre and post treatment with 4-OHA.

### Results

Nineteen patients were admitted to the trial. The age range was 58 to 83 years (average 68). All patients had previously undergone orchidectomy (13-33 months before entry: average 17) and 11 had been treated with AG and hydrocortisone (starting 4-13 months before entry: average 7). In all cases, AG and hydrocortisone was discontinued before entry. ECOG scores on entry ranged from 6 to 12.

Complete subjective response was seen in 4 (21%) and

**Table I** The Eastern Co-operative Oncology Group (ECOG) scale for subjective assessment

| | | | |
|---|---|---|---|
| *1. Pain* | | *2. Analgesic requirement* | |
| None | 0 | None | 0 |
| Slight/mild: little interference with non-strenuous activities | 1 | Non-narcotic occasional | 1 |
| Quite bad: interferes with daily activities and/or sleep | 2 | regular | 2 |
| Severe: distracted by pain much of the time | 3 | Narcotic occasional | 3 |
| Intolerable: dominates life | 4 | regular | 4 |
| *3. Performance status* | | | |
| Fully active | 0 | Scores are added: | |
| Active: capable of light work | 1 | Range is from | 0 (best) |
| Restricted: in bed <50% of the time; capable of self care | 2 | to | 12 (worst) |
| Restricted: in bed >50% of the time; limited self care | 3 | | |
| Bed-ridden | 4 | | |

partial response in 8 (42%), giving a total subjective response of 63%. In 10 of these 12 patients, the ECOG score at 4 weeks was 4 or less. Average duration of response was 10 weeks. No patient remained in remission for more than 16 weeks.

Side effects were few: 5 patients (31%) had a transient increase in bone pain following the first injection, necessitating an increase in analgesia. We attributed this to a 'tumour flare', the mechanism of which remains obscure. Most patients complained of pain at the injection site for 24–48 hours after each injection. In two cases this necessitated dose reduction but no patient had to discontinue treatment because of side effects.

In the 8 patients on whom endocrine studies were performed, 5 showed a significant decrease in oestradiol levels. There were no changes in testosterone, dihydrotestosterone or androstenedione levels.

### Discussion

Androgen deprivation has been the mainstay of treatment for advanced prostatic cancer since the pioneering studies of Huggins and Hodges (1941) on the effects of castration or oestrogen administration on men with prostate cancer. The use of anti-androgens (Scott & Schirmer, 1966) and LHRH analogues (Ahmed *et al*., 1985) have also been described. However, the role of oestrogens in prostatic biology is less well defined (for review see Mawhinney & Neubauer, 1979). It has been suggested that oestrogen suppression by aromatase inhibitors may be of benefit in benign prostatic hypertrophy (Henderson *et al*., 1986) although there is no experimental evidence as yet to implicate oestrogens in the causation or control of the malignant prostate.

Aromatase (oestrogen synthetase) is a key enzyme in the steroidogenic pathway from cholesterol to oestrogens. It mediates the conversion of androgens to oestrogens and is an enzyme complex involving NADPH, cytochrome c reductase and cytochrome p450. It is present in many tissues particularly placenta, breast, ovary, testes and adipose tissue. Its presence in prostate *in vitro* has been described (Stone *et al*., 1986) although it is still contentious (Smith *et al*., 1982). Aromatisation is the last reaction in the production of oestrogens and therefore blockage of this enzyme will not cause depletion of other steroids.

Selective inhibition of aromatase may be achieved by compounds which interfere with androgen aromatisation by binding to the enzyme, or by compounds, i.e. AG, which bind to cytochrome p450 and therefore act as competitive inhibitors. 4-OHA is a potent 'suicide inhibitor' of aromatase (Brodie *et al*., 1981) acting both by competition with the substrate and inactivation of the enzyme causing irreversible inhibition. Clinically, 4-OHA is in clinical trial use for the treatment of post-menopausal women with advanced breast cancer (Coombes *et al*., 1984).

All the patients in this study had 'end-stage' disease and we were struck by the quality of their responses, in marked contrast to those seen with AG/cortisone. Their feeling of well being was remarkable, and three of the patients were able to return to work for the duration of their remissions. Although subjective responses in such patients are open to criticism, the tumour 'flare' seen after the first injection suggests that there is a true biological response to aromatase inhibition. Additionally, the decrease in oestradiol levels observed in 5 of 8 patients in which it was measured lends support to this hypothesis.

This is the first study which describes the use of a selective aromatase inhibitor in patients with advanced, hormone insensitive, prostate cancer. We have recently commenced a clinical trial using 4-OHA in patients with advanced prostate cancer, which will include detailed objective measurements and endocrine analysis during treatment. We have also commenced laboratory studies to investigate the mechanism of action of 4-OHA in prostatic biology further.

4-OHA was supplied by Ciba-Geigy Pharmaceuticals, Horsham, West Sussex.

### References

AHMED, S.R., GRANT, J., SHALET, S.M. & 4 others (1985). Preliminary report of use of depot formulation of LHRH analogue ICI 118630 (Zoladex) in patients with prostatic cancer. *Br. Med. J.*, **290**, 185.

BRODIE, A.M.H., GARRET, W.M., HENDRICKSON, J.R., TSAI-MORRIS, C.H., MARCOTTE, P.A. & ROBINSON, C.H. (1981). Inactivation of aromatase in-vitro by 4-hydroxy-4-androstene-3-17-dione and 4-acetoxy-4-androstene-3-17-dione and sustained effects in vivo. *Steroids*, **38**, 693.

CHISHOLM, G.D. (1980). Prostate. In *Tutorials in Postgraduate Medicine, Urology*, Chisholm, G.D. (ed.) p. 223. Heinemann: London.

COOMBES, R.C., GOSS, P., DOWSETT, M., GAZET, J.-C. & BRODIE, A. (1984). 4-hydroxyandrostenedione in treatment of menopausal patients with advanced breast cancer. *Lancet*, **ii**, 1237.

DOWSETT, M., GOSS, P.E., POWLES, T.J. & 4 others (1987). Use of the aromatase inhibitor 4-hydroxyandrostenedione in post-menopausal breast cancer: optimization of therapeutic dose and route. *Cancer Res*., **47**, 1957.

DOWSETT, M., SHEARER, R.J., PONDER, B.A.J. & MALONE, P. (1988). The effects of aminoglutethimide and hydrocortisone, alone and combined, on androgen levels in post-orchiectomy prostatic cancer patients. *Br. J. Cancer*, **57**, 190.

HENDERSON, D., HABENICHT, U.-F., NISHINO, Y., KERB, B. & EL ETREBY, M.F. (1986). Aromatase inhibitors and benign prostatic hypertrophy. *J. Steroid Biochem.* **25**, 867.

HUGGINS, C. & HODGES, C.V. (1941). Studies on prostate cancer. i. The effects of castration, or oestrogen and of androgen injection on serum phosphatases in metastatic carcinoma of the prostate gland. *Cancer Res*., **1**, 293.

LANCET (1980). Cancer of the prostate. *Lancet*, **ii**, 1009.

MAWHINNEY, M.G. & BLAKE, N.L. (1979). Actions of oestrogens in the male. *Invest. Urol*., **16**, 409.

PARKER, M.C., COOK, A., RIDDLE, P.R., FRYATT, I., O'SULLIVAN, J.P. & SHEARER, R.J. (1985). Is delayed treatment justified in carcinoma of the prostate? *Br. J. Urol*., **57**, 724.

PONDER, B.A.J., SHEARER, R.J., POCOCK, R.D. & 4 others (1984). Response to aminoglutethimide and cortisone acetate in advanced prostate cancer. *Br. J. Cancer*, **50**, 757.

PLOWMAN, P.N., PERRY, L.A. & CHARD, T. (1987). Androgen suppression by hydrocortisone without aminoglutethimide in orchiectomised men. *Br. J. Urol*., **59**, 255.

SCOTT, W.W. & SCHIRMER, H.K. (1966). A new oral progestational steroid effective in treating prostatic cancer. *Trans. Am. Assoc. Genitourin. Surg*., **58**, 54.

SMITH, T., CHISHOLM, G.D. & HABIB, F.K. (1982). Failure of human benign prostatic hyperplasia to aromatise testosterone. *J. Steroid Biochem*., **17**, 119.

STONE, N., FAIR, W.R. & FISHAM, J. (1986). Oestrogen formation in human prostatic tissue from patients with and without benign prostatic hypertrophy. *Prostate*, **9**, 311.

WORGUL, T.J., SANTEN, R.J., SAMOJILK, E., VELDHUIS, J.D., LIPTON, A. & HARVEY, H.A. (1983). Clinical and biochemical effects of aminoglutethimide in the treatment of advanced prostate cancer. *J. Urol*., **129**, 51.